# Probability of early infection extinction depends linearly on the virus clearance rate

**DOI:** 10.1098/rsos.240903

**Published:** 2024-10-02

**Authors:** N. Juhász, F. A. Bartha, S. Marzban, R. Han, G. Röst

**Affiliations:** ^1^ National Laboratory for Health Security, 6720 Szeged, Hungary; ^2^ Bolyai Institute, University of Szeged, 6720 Szeged, Hungary; ^3^ Integrated Mathematical Oncology Department, H. Lee Moffitt Cancer Center and Research Institute, Tampa, FL, USA; ^4^ School of Sciences, Zhejiang University of Science and Technology, Hangzhou, 310023, People’s Republic of China; ^5^ Hungarian Centre of Excellence for Molecular Medicine (HCEMM), Szeged, Hungary

**Keywords:** agent-based models, multiscale mathematical modelling, SARS-CoV-2, branching processes, stochastic extinction, spatially explicit model

## Abstract

We provide an *in silico* study of stochastic viral infection extinction from a pharmacokinetical viewpoint. Our work considers a non-specific antiviral drug that increases the virus clearance rate, and we investigate the effect of this drug on early infection extinction. Infection extinction data are generated by a hybrid multiscale framework that applies both continuous and discrete mathematical approaches. The central result of our paper is the observation, analysis and explanation of a linear relationship between the virus clearance rate and the probability of early infection extinction. The derivation behind this simple relationship is given by merging different mathematical toolboxes.

## 1. Introduction

With emerging new variants and waning of immunity, SARS-CoV-2 infection continues to be a relevant problem worldwide. There is an important need to develop a comprehensive understanding of in-host viral dynamics and antiviral therapies in particular.

Mathematical models have been indispensable tools in tackling the challenges posed by the appearance of the novel coronavirus SARS-CoV-2. From assessing the effects of a nationwide lockdown to predicting the immune response in the lung, there is a great variety of mathematical frameworks targeting various aspects of COVID-19. The present article focuses on in-host, cellular level *in silico* examination. Most mathematical modellers who investigate within-host SARS-CoV-2 infection apply ordinary differential equations [1–7]; however, more complex approaches have been employed as well. As an example for the latter, we refer to the data-based approach suggested in [8], and the multiscale system proposed in [9]. The present study uses a hybrid multiscale approach as well.

Hybrid mathematical models are applied for a wide range of phenomena in life sciences [10,11]. From investigating the mechanical properties of bone osteoblasts [12] to assessing the pharmacometrical properties of an antiviral drug [13], multiscale frameworks are a promising option for a variety of reasons. This system is suitable to model within-host, cellular level virus dynamical phenomena with a special attention to the significantly different scales of interacting entities (i.e. epithelial cells and viruses). Conceptually, the fundamental idea behind such hybrid systems is to form bridges between two modelling strategies, hence uniting the respective advantages of discrete and continuous techniques. We will heavily rely on the model developed in [14] both to investigate the offspring distribution of infected cells and to assess early infection extinction.

Virus infections are inherently stochastic. Even though this probabilistic property remains throughout the entire course of the process, it is the early phase when the variability in qualitative behaviour is largest. In particular, not all inoculation events lead to established infection. Even when the basic reproduction number $R_{0}>1,$ initial virus spread can die out stochastically. The extinction problem was investigated relatively widely for HIV infection. The authors of [15–17] used Monte Carlo approaches to examine virus elimination, the work of Merrill [18] modelled the initial phase of infection as a branching process, while [19,20] described early infection as a diffusion process. The present study also focuses on the extinction problem, but we use a different mathematical approach: our principal model is a multiscale framework. Our main goal is to identify and explain the relationship between the internal virus clearance rate and the probability of early infection extinction within the framework of a relatively complex, hybrid mathematical model. We highlight the work of Pearson *et al*. [21] where the authors use a transition model and the Gillespie algorithm to investigate a similar problem. Their model does not consider spatial factors and it allows for the derivation of a closed formula for the probability of extinction. A similar solution is not feasible in our spatially explicit model. We focus on obtaining and explaining an analogous relationship as shown in [21], but within the framework of our hybrid mathematical system.

In our study, we will apply a combination of different mathematical toolboxes. Synthetic data are generated by a hybrid multiscale system, our observations are explained by means of the theory of branching processes and a simple viral kinetic model is used for comparison as well.

Our main goal here is to uncover new underlying features of the early phase of SARS-CoV-2 infection, to study the role of offspring distribution in the outcome and to explore the impact of prophylactic antivirals.

## 2. Methods

In the following, we describe the three main mathematical frameworks used in this study, i.e. the target cell limited model, the hybrid agent-based model (ABM)–partial differential equation (PDE) system and an approach using branching processes. We emphasize that each of these three models is a whole on its own, and each system is defined separately and independently from the other two: these frameworks model the same real-life process, and none of them is part of another. We also note that each model focuses on different aspects of infection spread and they use substantially distinct modelling assumptions.

### 2.1. Target cell limited model

The standard target cell limited model is defined by the following set of ordinary differential equations [22]:


(2.1) $$\left\{ \begin{array}{l} \frac{dT(t)}{dt}=-\beta T(t)V(t),\\ \frac{dI(t)}{dt}=\beta T(t)V(t)-\delta I(t),\\ \frac{dV(t)}{dt}=pI(t)-cV(t),\\ \frac{dD(t)}{dt}=\delta I(t). \end{array}\right.$$

In this classical viral kinetic model, the functions T(t),I(t),V(t),D(t) describe the number of target cells, the number of infected cells, the total number of virions and the dead cells at time t≥0, respectively. Concerning model parameters, β represents the target cells’ infection rate, δ denotes the death rate of infected cells, p stands for the virus production rate and c is the virus clearance rate. The basic reproduction number of the above viral kinetic model is given by


(2.2) $$R_{0}=\frac{p\beta T_{0}}{c\delta }.$$

This is crucial in our case as it establishes a connection between the virus clearance rate and the average number of new infections due to a single infected individual, namely


(2.3) $$c∼\frac{1}{R_{0}}.$$

That is, c is inversely proportional to $R_{0}.$

### 2.2. The hybrid PDE–ABM model

The main hybrid framework is defined via forming bridges between two significantly different modelling approaches: agent-based models and partial differential equations.

Let Ω denote the spatial domain we are modelling. For an *in vitro* experiment, this would be the area of a single well in a laboratory plate, while in the case of an *in vivo* process, it could represent a small part of the lung tissue.

Section 2.2.1 introduces the discrete component of our multiscale framework describing epithelial cells, whereas §2.2.2 discusses the continuous part modelling the virus concentration V. The fundamental observation suggesting this separation is that epithelial cells are several magnitudes larger than virus particles [23–25].

Parametrization, initial conditions and further details of the model are given in §2.2.3 and in our previous work [14].

#### 2.2.1. Epithelial cells

Following [14], epithelial lung cells are defined as discrete agents in an ABM. The discrete state space of target cells is constructed exactly as in [14]; here we only recall the most fundamental technical details. We define a two-dimensional ABM state space by introducing a grid of $k_{1}\times k_{2}$ agents representing lung cells ($k_{1},k_{2}\in \mathbb{N}$). Epithelial cells are identified by the corresponding agent’s place in the lattice, or formally, by the (i,j) indices, where $(i,j)\in J=\{(i,j)|1\leq j\leq k_{2},$$1\leq j\leq k_{2}\}$. Finally, by setting the $\Omega_{i,j}$ notation for the open set occupied by the (i,j)th cell, we have $\bar{\Omega }=\underset{(i,j)\in J}{⋃}{\bar{\Omega }}_{i,j}$.

Concerning cell states within the ABM space, the main idea is straightforward: each agent has three potential states. The latter concept is formally grasped by the state function $s_{i,j}(t)$ representing that an epithelial lung cell is considered to be either a target cell, an infected cell or a dead cell in our framework:


$$\begin{array}{r} s_{i,j}(t)=\left\{ \begin{array}{ll} \text{T}, & \text{if the }\left(i,j\right)\text{th cell is alive and uninfected at time }t\text{,}\\ \text{I}, & \text{if the }\left(i,j\right)\text{th cell is infected at time }t\text{,}\\ \text{D}, & \text{if the }\left(i,j\right)\text{th cell is dead at time }t\text{.} \end{array}\right. \end{array}$$

We note that the virological terminology for a cell susceptible to virus infection is *target cell* [22].

Regarding state dynamics, the transition rules are defined to mimic the acute infection process we model. the complete list is as follows:

— The time frame of infection is relatively short, hence cell apoptosis and cell division are ignored.
— Infection is not reversible: once a cell gets infected, it cannot become a functioning target cell once again.
— Viral infection is the only cause for cell death.
— Infected cells eventually die.
— The *target cell *→ *infected cell* state change: a target cell may become infected depending on the local virus concentration at the given cell’s location. Infection is randomized and it occurs with a probability of $P_{I}$, depending on the virus concentration (for more details, see [14]).
— The *infected cell *→ *dead cell* state change: analogously to infection, cell death occurs stochastically with probability $P_{D}$.

#### 2.2.2. Virus concentration

In the second part of the framework, we define virus concentration v=v(t,x,y) as a variable that is continuous in both space and time, and as such, its dynamics is described by the partial differential equation


$$\frac{\partial v(t,x,y)}{\partial t}=D_{v}\Delta v-c\cdot v(t,x,y)+\sum_{(i,j)\in J}g_{i,j}(t,x,y),\;t>0,\;\;(x,y)\in \Omega ,$$

where $D_{v}$ denotes the virus diffusion coefficient in units [(14 μm)^2^ min^−1^], c stands for the virus clearance rate in units [min^−1^], while $g_{i,j}$ represents the viral source term in units [copies (ml min cell)^−1^] for the (i,j)th cell. We note that the unit (14 μm)^2^ min^−1^ is the result of the spatial discretization resolution we apply in our PDE–ABM model, where this particular resolution was directly motivated by the physical dimension of an epithelial cell. As for boundary conditions, we assume zero flux across the edges of the domain, hence we apply $\partial v/\partial \vec{n}=0$ at the boundary. The above equation captures the general concept that virus particles spread across the domain via diffusion, the immune system removes viruses at a constant rate, while new virions are generated by infected cells in a process grasped by the $g_{i,j}$ source functions:


(2.4) $$\begin{array}{r} g_{i,j}(t,x,y)=\left\{ \begin{array}{ll} 0, & \text{if }s_{i,j}(t)=\text{T}\text{ and }(x,y)\in \Omega_{i,j},\\ f_{i,j}(t,x,y), & \text{if }s_{i,j}(t)=\text{I}\text{ and }(x,y)\in \Omega_{i,j},\\ 0, & \text{if }s_{i,j}(t)=\text{D}\text{ and }(x,y)\in \Omega_{i,j},\\ 0, & \text{if }(x,y)\notin \Omega_{i,j}. \end{array}\right. \end{array}$$

In general, any reasonable $f_{i,j}(t,x,y)$ function may be allowed in the formula above (for more details, see [14,26]). In particular, we used the standard simplification commonly applied in the field of viral dynamics: similarly to [27,28], a constant virus budding rate is assumed. Specifically, we used the estimate $f_{i,j}$ = 3.72 × 10${,}^{-3}$ [copies (ml min cell)^−1^] (see [29]).

The multiscale model becomes complete by explicitly taking into account the interactions each component-system has with the other. The discrete ABM part tangibly affects the PDE describing virus concentration through the $g_{i,j}$ source functions; on the other hand, the continuous viral part makes a difference within the agent-based subsystem through affecting the local probability of the *target cell *→* infected cell* state change.

This framework has two fundamentally important advantages. On the one hand, it yields spatially explicit information on virus propagation patterns, and on the other hand, its stochastic approach to state changes supports realistic simulation outputs. In more detail, the latter means that both the infection process (i.e. the *target cell *→* infected cell* state change) and cell death itself (i.e. the *infected cell *→ *dead cell* state change) are internally designed as non-deterministic processes: their implementation reflects the natural randomness of these events and the variability of outcomes.

#### 2.2.3. Implementation and main setting

Our original implementation of the hybrid PDE–ABM system (including the discussion of the default parameters) is described in detail in [14]. Each simulation follows a total number of 40 000 cells in a grid of dimensions 200×200 over the course of a SARS-CoV-2 infection process.

The main difference between the present implementation and that described in [14] is that in this investigation infection always starts from a single infected cell. Specifically, every simulation begins with the deterministic initial condition


(2.5) $$I_{0}=1,\;T_{0}=39\;999,\;v(0,x,y)=0,$$

where I denotes the number of infected cells, T stands for the number of target cells and v(t,x,y) is the virus concentration. In each simulation, the initial infected cell is placed in the centre of the cell lattice.

Technically, there are two different execution modes in our implementation, which allow us to investigate two separate aspects of the infection process. The first mode essentially corresponds to the natural course of virus infection: all infected cells produce virions, which, in turn, may infect further target cells. This mode is suitable to determine the empirical probability of extinction. The second mode was implemented to assess the effect associated with a single infected cell, i.e. in order to investigate the offspring distribution and the $R_{0}$ value. In this second mode, the only cell that is able to produce virions is the sole original infected cell we focus on, while virus production is disabled for all further infected cells. This artificial setting allows us to track the number of new infections generated by a single infected cell. Further details are given in the Github repository [30].

Our implementation of the proposed hybrid system is based on an adaptation of the free and open source package HAL (Hybrid Automata Library) [31]. The updated software carrying out these new *in silico* experiments is a straightforward modification of our original implementation and is publicly available on Github [30].

The offspring distribution histograms and other related calculations were completed using Wolfram Mathematica [32].

### 2.3. Discrete-time branching processes

The theory of branching processes provides another classical model to mathematically describe virus infection. Branching processes [33] have been applied to neutron chain reactions, cancer growth, the survival of mutant genes and population growth. One of its main concerns is population extinction due to randomness.

Let $X_{n}$ denote the total size of the nth generation ($n\in {\mathbb{N}}_{0}$) within the population—in our case, the infected cells. We note that a cell is in the nth generation if it was infected by a virion that had been produced by an infected cell in the (n−1)th generation.

The process $\{X_{n}{\}}_{n=0}^{\infty }$ is called a Galton–Watson branching process if the following three conditions hold.

1. Each individual in generation n has $Y_{n}$ offspring in the next generation, where $Y_{n}$ is a random variable that takes values in ${\mathbb{N}}_{0}.$ The offspring distribution of $Y_{n}$ is $\{p_{n,k}{\}}_{k=0}^{\infty },$ i.e. we have $P(Y_{n}=k)=p_{n,k},\;k=0,1,2,\ldots$
2. The number of offspring an individual has is independent from all other individuals of the population.
3. All generations share the same offspring distribution, i.e. we have $Y_{n}\equiv Y\equiv \{p_{n,k}{\}}_{k=0}^{\infty },$ where $n\in {\mathbb{N}}_{0}.$

We recall that the probability of population extinction


$$\lim_{n\to \infty }\;P(X_{n}=0)$$

is given by the solution of a fixed-point problem. Specifically, assuming that the probability generating function $G_{Y}$ of the offspring distribution $\{p_{k}{\}}_{k=0}^{\infty }$ satisfies certain general properties (for more details, see [33]); moreover, $X_{0}=1,$ and 𝔼(Y)>1; then there exists a unique q∈(0,1), such that $G_{Y}(q)=q.$ Then,


(2.6) $$\begin{array}{ccc} & q=\lim_{n\to \infty }\;P(X_{n}=0) & \end{array}$$

readily follows. That is, even for a Galton–Watson process where the expected value of the offspring distribution is larger than 1, the probability of extinction q is strictly larger than 0.

## 3. Results

### 3.1. Observed probability of extinction

This section investigates the empirical probability of infection extinction (POE) as a function of the virus clearance rate. Specifically, starting from the virus clearance constant of SARS-CoV-2 [14], i.e.


(3.1) $$c=c_{SC2}=1.67\times 10^{-3}\;[{min}^{-1}],$$

we loop through increasing virus clearance parameter values of c mimicking the effect of an antiviral drug. For each value of c, we execute 1000 randomized simulations in our hybrid framework. A simulation’s outcome classifies as extinct if at least 99% of target cells remain uninfected in the end.

As we mentioned in §2.2.3, we use two different modes of infection implementation. Here we apply the version that corresponds to the natural course of virus infection: when a cell gets infected it starts producing virions, which in turn will infect further target cells, and so on. This is naturally suitable to determine the empirical POE.

**Figure 1.**
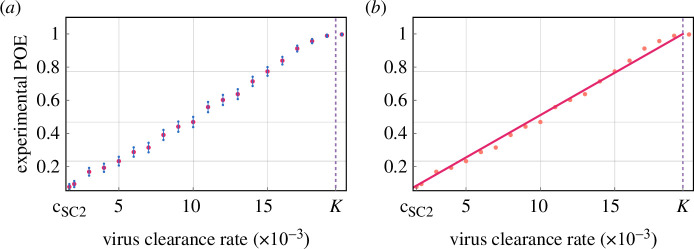
(*a*) *In silico* empirical POE values generated by the hybrid PDE–ABM model for increasing virus clearance rates. A 95% confidence interval is shown around each estimated POE value. The intervals were calculated with the formula $\bar{p}\pm z_{\alpha }\cdot \sqrt{\bar{p}(1-\bar{p})}/\sqrt{1000},$ where $\bar{p}$ is the empirical POE and $z_{\alpha }$ is the corresponding quantile of the standard normal distribution. (*b*) The same data points as given in (*a*) shown against the best linear fit.


A visual representation of our results is given in figure 1; the data points resemble a straight line. Calculating the best linear fit yields y=0.051⋅x with an $R^{2}$ value of 0.994, the fitted line attaining the value 1 at approximately

$$c=19.5854\times 10^{-3}\;[{\text{min}}^{-1}]:=K.$$

**Figure 2.**
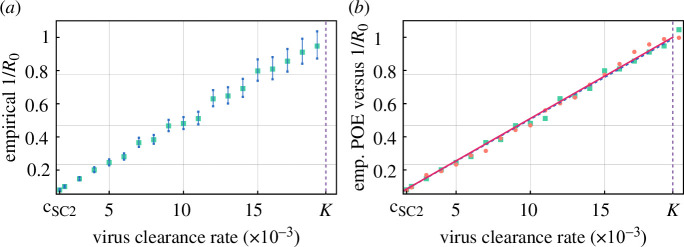
(*a*) Empirical values of $1/R_{0}$ clearly follow a linear trend. The 95% confidence intervals are shown in blue. (*b*) Our empirical POE results (pink dots) and the corresponding fitted line (in magenta) shown against empirical $1/R_{0}$ values (green squares) and the corresponding fitted line (in blue). Note that the blue line is so close to the magenta line that it is barely visible.

The probability of extinction was investigated in [21] as well, where the authors found that in their transition model the POE can be formulated as $\min(1/R_{0},1).$ In order to compare the respective results, we calculated the empirical $R_{0}$ values corresponding to each c value via generating *in silico* data by the PDE–ABM model, using the aforementioned second mode in the implementation. In figure 2*b*, we depict both (i) our results for the empirical POE and (ii) the empirical $1/R_{0}$ values. The outcome in figure 2*b* illustrates an obvious correspondence between our empirical POE values and the respective closed formula of $1/R_{0}$ in [21] as the two sets of data points are clearly scattered along the same line. This is consistent with the result of a *t*‐test for the difference values, where we obtain p=0.71 for the test’s *p*‐value. For reference, the line fitted to the empirical $1/R_{0}$ data is given by y=0.05056⋅x, the $R^{2}$ value in this case being 0.9954. In conclusion, our results match the formula shown in [21].

Another important consequence of figure 2 is that


(3.2) $$c∼\frac{1}{R_{0}}$$

holds in our hybrid mathematical framework.

Note that this result, now obtained for the PDE–ABM model, is consistent with the well-known formula for $R_{0}$ in the target cell limited model; see (2.3).

To summarize, the empirical POE values seem to follow a linear trend in our framework. The subsequent sections focus on finding a mathematical explanation for our observations.

### 3.2. Theoretical probability of extinction

We recall that a closed formula was derived for an analogous linear relationship in [21], but our spatially explicit hybrid system does not allow for a similar explanation.

We begin by addressing the question as to whether this fundamental dynamical behaviour in our *in silico* experiments may be approached with a branching process.

First, for most values of $c\in [c_{SC2},K]$ (i.e. unless c takes an extremely high value), infection either dies out almost immediately or it blows up exponentially after the first few steps—in other words, spontaneous extinction virtually never happens once 5–10 cells have been infected. This means that, from the viewpoint of the probability of extinction, the focus is almost exclusively on the beginning of infection:


$$\begin{array}{r} \begin{array}{rl} \text{POE}\equiv P( & \text{spontaneous extinction (ever)})\approx \\ & P(\text{spontaneous extinction in the very beginning}). \end{array} \end{array}$$

Second, the saturation of the cell state space is not a significant obstacle, the condition regarding independent offspring distributions is not violated: in the initial phase of the infection process there are only a few infected cells, virions are able to move due to natural diffusion and, hence, infections in these first few iterations are approximately independent.

Thus, from this point, we study the number of infected cells in the form of a discrete-time Galton–Watson process $\{X_{n}{\}}_{n=0}^{\infty }$.

#### 3.2.1. Offspring distribution

The number of direct successors or offspring of one single cell (i.e. the number of new infections generated by the latter) is captured by the random variable Y in the infection’s branching process representation. In order to apply the fixed-point theorem (2.6), we need to establish a precise understanding of the distribution $\{P(Y=k){\}}_{k=0}^{\infty }\equiv \{p_{k}{\}}_{k=0}^{\infty }$.

We focus on the generation change from $X_{0}$ to $X_{1},$ where the number of originally infected cells $X_{0}$ is set to 1 for simplicity. The offspring distribution of a single infected cell within our hybrid PDE–ABM simulations can be approximated by means of frequency histograms. Specifically, we estimate the $\{p_{k}{\}}_{k=0}^{\infty }$ probabilities by generating an adequate histogram for each virus clearance value. The histograms are calculated by executing 1000 randomized simulations for each value of c and examining how many direct successors are generated by the sole original infected cell.

We make a few technical remarks for completeness:

— Note that even though these simulations are not stopped at any pre-defined constant time, within the context of the branching process they still represent only one generation step.
— In order to model the early phase of infection and, in particular, the offspring distribution during early infection, we use the previously mentioned second mode of our program code. In this case, newly infected cells do not produce virions, hence every new infection is the direct successor of the sole infected cell present at the 0th generation.

**Figure 3.**
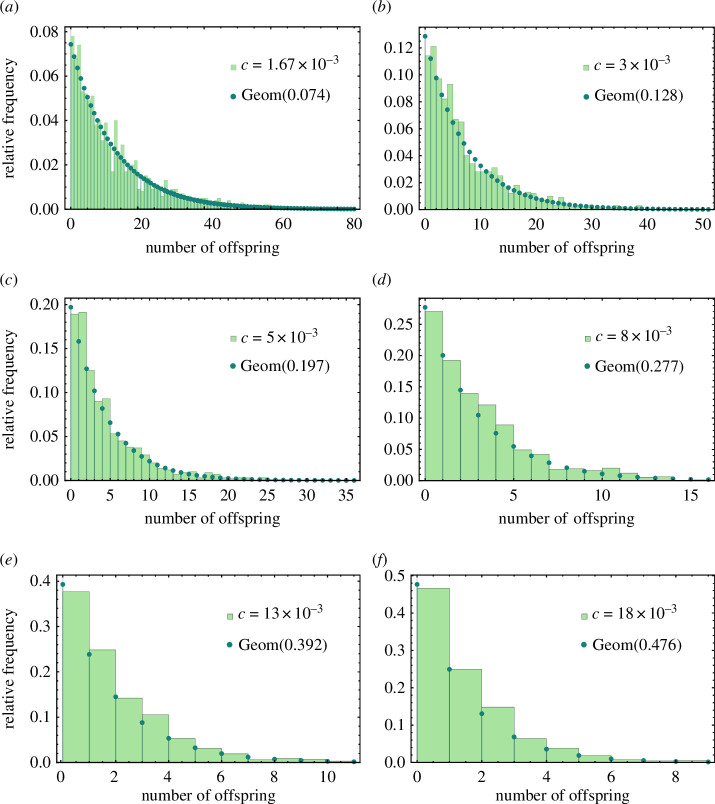
Empirical frequency histograms calculated to estimate the offspring distribution Y (bars filled with light green) are shown against best geometric fit (dots depicted with dark green). Each histogram is a result of 1000 randomized simulations in our PDE–ABM framework, and simulations for a single histogram were executed with a specific, constant virus clearance rate c. All simulations were initialized with a single infected cell in the centre of the cell grid and were executed with otherwise identical SARS-CoV-2 parameters (except for the value c, naturally).

Our results are depicted in figure 3. The histograms show a consistent similarity to geometric distribution for any virus clearance rate $c\in [c_{SC2},K]$.

Hence, from now on, we assume Y∼Geom(λ), where λ=λ(c).

#### 3.2.2. Parameter fit

In our case, Y is supported on ${\mathbb{N}}_{0}$ (note not on ${\mathbb{N}}^{+}$); hence, the maximum likelihood estimate (MLE) of the geometric distribution’s parameter is


(3.3) $$\hat{\lambda }=\frac{1}{1+\bar{E}(Y)},$$

where $\bar{E}(Y)$ denotes the sample mean for Y.

**Figure 4.**
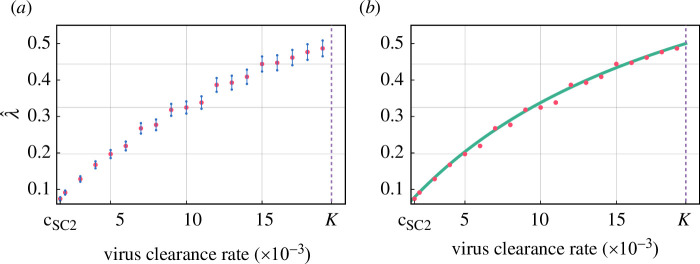
(*a*) Maximum likelihood estimates of λ in Y∼Geom(λ). Each $\hat{\lambda }$ value was calculated based on 1000 randomized simulation outputs computed in our PDE–ABM framework. A 95% confidence interval is shown around each estimated $\hat{\lambda }$ value. The intervals were calculated using the Fisher information formula. (*b*) The same MLE values as given in (*a*) shown against the Hill curve H(x).


We visualize these MLE values for λ as a function of c in figure 4*a*. There is a reasonably good correspondence between our data points and a Hill equation with n=1 as exponent (the latter is motivated by equations (3.2) and (3.3)). Figure 4*b* shows the $\hat{\lambda }$ values against the Hill curve defined by

$$H(x)=\frac{1}{1+\frac{K}{x}},$$

where K is as in §3.1.

Our results suggest choosing the parameter λ=λ(c) of the approximating geometric distributions in the form of


(3.4) $$\begin{array}{ccc} & \lambda (c)=\frac{1}{1+\frac{K}{c}}. & \end{array}$$

#### 3.2.3. Explaining the observed linearity

Here we show that in this modelling framework, the POE as a function of c is indeed linear.

The method we present here is based on equation (2.6). That is, the probability of extinction q is the fixed point of the probability generating function


(3.5) $$\begin{array}{ccc} & G_{Y(\lambda )}(q)=\frac{\lambda }{1-(1-\lambda )q}=q. & \end{array}$$

**Figure 5.**
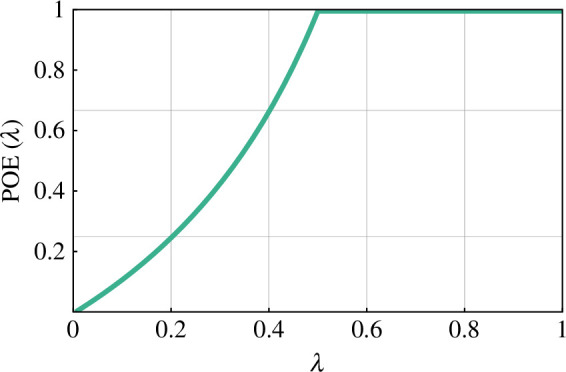
The probability of infection extinction as a function of λ, obtained as a solution of the fixed-point equation (2.6).

Consequently, we have


(3.6) $$\begin{array}{ccc} & \lambda =q\cdot (1-(1-\lambda )q), & \end{array}$$

yielding the POE as the non-negative solution


(3.7) $$\begin{array}{ccc} & \text{POE}(\lambda )=\frac{1-\sqrt{1-4\cdot (1-\lambda )\cdot \lambda }}{2\cdot (1-\lambda )}, & \end{array}$$

depicted in figure 5.

It is straightforward to check that c↦POE(λ(c)) is a linear function of c by substituting (3.4) into (3.7).

This connection is guaranteed by three factors in our *in silico* experiments. Firstly, the complex infection dynamics emerging in the PDE–ABM framework appears to lead to a geometric offspring distribution in the initial phase of the infection process; secondly, the free parameter in our observations is the virus clearance rate c; and thirdly, $c∼\frac{1}{R_{0}}$ holds. Clearly, these three conditions lead to equations (3.4) and (3.7) and their composition results in a linear function.

Finally we note that by substituting λ=1/(1+𝔼(Y)) into (3.7), we directly obtain POE=1/𝔼(Y). Consequently, we have $\text{POE}=1/R_{0}$ in the branching process framework, corresponding to the respective extinction formula obtained in [21].

## 4. Discussion

In this work, we used the combination of different mathematical approaches to generate, observe and explain a specific phenomenon in the early phase of virus infection.

Our results may be divided into two main parts. First, we focused on generating a large quantity of adequate *in silico* empirical data with a hybrid mathematical framework (the PDE–ABM model), while the second component of our work consisted in establishing a theoretical explanation for the behaviour of this data using the theory of branching processes.

We considered a non-specific prophylactic antiviral drug that increases the internal virus clearance rate in the body, generated respective infection extinction data and investigated the drug’s role in the outcome. We observed clearly linear trends emerging for both the empirical POE and $1/R_{0}$ with respect to the internal virus clearance parameter c.

An important result and novelty of our paper is the theoretical explanation we provide for the emerging linearity. We successfully estimated the offspring distribution of the branching process describing early-phase virus spread, based purely on the original model assumptions and our computer-generated data. We concluded that the number of new infections generated by a single infected cell is described well by a geometric distribution. Moreover, we found both that the relationship between the parameter λ of the geometric distribution and virus clearance rate is described by a Hill function, and that it is precisely this latter function that linearizes the non-negative solution of the fixed-point problem.

The second main result of this paper is the detection of a good correspondence overarching a variety of mathematical models; namely, the hybrid PDE–ABM system, the branching process theory, and the transition model used in [21]. The visualization of our *in silico* data shows that the probability of infection extinction in the hybrid model corresponds to the formula $1/R_{0}$ obtained in [21] for a not spatially explicit transition model, while we analytically verified the same equation for the branching process theory as well. With this we showed that the branching process theory captures well the hybrid system’s initial behaviour. Our results also demonstrate that the respective formula and dynamics obtained in [21] are not an artefact of their specific mathematical approach; on the contrary, it seems to be a general phenomenon that is present in substantially different models.

Concerning the limitations of our work, we note that directly increasing the virus clearance rate c does not strictly correspond to increasing drug dose; this relationship can be quite involved. We also emphasize that the biological implications of our results are applicable and relevant only for *in vitro* experiments, or prophylactically administered drugs, when the drug is present already at the initiation of infection.

Finally, we highlight that our findings imply that a similar linearity property is to be expected in various other natural spreading phenomena whenever the underlying offspring distribution is geometric and the free parameter is proportional to $1/R_{0}.$

## Data Availability

Data and relevant code for this research work are stored in GitHub [30] and have been archived within the Zenodo repository [34]. Supplementary material is available online [35].
